# Evaluation of LN34 real-time PCR assay for detection of rabies virus in human and animal specimens

**DOI:** 10.1515/almed-2025-0197

**Published:** 2026-07-07

**Authors:** Lonika Lodha, Manoor Ananda Ashwini, Sathya Priya Manuel, Arpita Maladakar, Shubhangi Chandel, Preeti Soundarya Bhalke, Shrinivas Navya, Kuntanahalli Narayanaswamy Krupa, Shrikrishna Isloor, Balaji Chandrashekar, Anita Mahadevan, Reeta S. Mani

**Affiliations:** Department of Neurovirology, National Institute of Mental Health and Neurosciences (NIMHANS), Bengaluru, India; Department of Microbiology Veterinary College, KVAFSU-CVA Rabies Diagnostic Laboratory, WOAH Reference Laboratory for Rabies, KVAFSU, Bengaluru, India; Mission Rabies-WVS, Goa, India; Department of Neuropathology, National Institute of Mental Health and Neurosciences (NIMHANS), Bengaluru, India

**Keywords:** rabies, zoonoses, laboratory diagnosis of rabies, molecular diagnosis, one health

## Abstract

**Objectives:**

Rabies, a neglected zoonotic encephalitis caused by lyssaviruses, claims around 59,000 lives annually. PCR-based methods are pivotal for laboratory confirmation of rabies in humans and animals. The LN34 pan-lyssavirus real time PCR assay has demonstrated good diagnostic performance in detecting rabies viral RNA in extensive evaluations with animal brain tissues. However, its application has not been adequately evaluated on human samples.

**Methods:**

This study assessed the utility of the LN34 assay on human brain tissues, and antemortem samples such as cerebrospinal fluid, saliva and nuchal skin. Furthermore, this assay was also evaluated using postmortem dog saliva samples, in addition to animal brain samples.

**Results:**

Compared to the gold-standard fluorescent antibody test (FAT), the LN34 assay showed 100 % sensitivity and specificity for both human and animal brain samples. For human antemortem samples, there was perfect agreement between the LN34 assay and routine real-time RT-PCR for detection of rabies virus (RABV). Similarly, there was complete agreement between the two assays when testing dog saliva samples. The sensitivity and specificity of saliva LN34 assay in laboratory-confirmed rabid dogs was 50 and 100 % respectively.

**Conclusions:**

The LN34 assay is a suitable alternative for diagnosis and surveillance of rabies in humans and animals in an area endemic for rabies lyssavirus.

## Introduction

Rabies is caused by viruses of the genus *Lyssavirus*, most importantly the rabies lyssavirus [1]. It is a universally fatal zoonotic encephalitis transmitted through exposures to rabid animals, and responsible for approximately 59,000 deaths annually worldwide [2]. Only rabies lyssavirus is known to occur in India, and the predominant mode of transmission is dog-mediated, with other mammals responsible for occasional cases.

There is no conclusive evidence of bat-mediated transmission of human rabies in India, although serological evidence of lyssavirus infection has been demonstrated in bats [3], and non-rabies lyssaviruses have been reported in bats in this region [4].

In both animals and humans, postmortem diagnostic confirmation of rabies is achieved by antigen detection using direct fluorescent antibody test (FAT), which is the current gold standard. Other tests such as direct rapid immunohistochemistry test (dRIT), or detection of rabies viral nucleic acid from brain samples can also be used [5]. Additionally, in humans, antemortem confirmation of rabies relies on detection of viral antigens (from nuchal skin biopsy) or nucleic acids [from cerebrospinal fluid (CSF), saliva, and nuchal skin biopsy], detection of neutralizing antibodies in serum and CSF, and viral isolation from biological fluids [6].

FAT is inherently challenging due to the necessity of virus-positive controls, procurement and maintenance of expensive equipment and consumables, and subjectivity in interpretation [7], 8]. Serology is not useful in the diagnosis of animal rabies, and its interpretation in human samples is dependent on an accurate vaccination history, which may not be possible to obtain. Thus, PCR is indispensable for both antemortem (in humans) and postmortem (in humans and animals) diagnosis of rabies, due to its high diagnostic yield and objective interpretation.

As real-time PCR utilizing Taqman chemistry is the currently preferred molecular test for rabies, the LN34 assay, which is a pan-lyssavirus real-time PCR, was evaluated in this study. The assay utilizes a combination of degenerate primers and a multiplexed, locked nucleic acid (LNA) Taqman probe to increase the broad reactivity of the assay [9]. It has been reported to have high diagnostic specificity (99.68 %) and sensitivity (99.90 %) when compared to FAT [9], and can detect 13 non-rabies lyssaviruses, and a large number of rabies virus variants [10]. The use of a pan-lyssavirus PCR for surveillance of rabies is important to detect the emergence of any non-rabies lyssavirus in India. Although the excellent diagnostic performance of this assay is well-documented for animal brain samples, it has not been tested on a large scale with human postmortem or antemortem samples, and it has also not been used on animal saliva samples.

Therefore, the aim of this study was to evaluate the pan-lyssavirus LN34 multiplex real-time reverse transcriptase PCR assay in a tertiary level virology laboratory situated in Southern India. The LN34 assay was compared with the routinely used RT-PCR assay which targets the N gene of the rabies lyssavirus [11].

## Materials and methods

### Study design and specimens

This study was an observational, laboratory-based study, carried out at the WHO Collaborating Center for Reference and Research on Rabies situated at the Department of Neurovirology, National Institute of Mental Health and Neurosciences, in Bangalore in Southern India.

The samples included in this study were postmortem brain tissues from animals obtained through occipital foramen approach, and postmortem saliva as oral swabs in viral transport medium from dogs. From humans, postmortem brain tissue in glycerol saline, and antemortem CSF, pooled saliva aspirated from the corner of the mouth, and nuchal skin samples were transported in sterile containers without any preservative, under cold chain. Antemortem samples from humans were obtained at varying stages of disease. Brain tissues were subjected to FAT and LN34 assay. Antemortem human samples, and dog saliva samples were tested with the routine real-time RT-PCR assay, and LN34 assay.

### Fluorescent antibody test (FAT)

The fluorescent antibody test (FAT) for rabies nucleoprotein antigen detection, which is the current gold standard, was performed on all human and animal brain tissues, as previously described [12]. Briefly, cut surfaces of fresh brain tissues were used to make smears, which were then fixed in cold acetone for 2 h, air dried and treated with a cocktail of anti-rabies monoclonal antibodies conjugated with FITC (EMD Millipore Corporation, Temecula, CA) for 30 min at 37 °C in an incubator in a humid chamber. Known rabies positive and negative brain smears were included as controls. The slides were examined under a fluorescence microscope.

### Real-time RT-PCR for detection of rabies RNA

A commercially available kit, i.e., i.e., QIAamp Viral RNA minikit (Qiagen, Hilden, Germany), was used to extract nucleic acids from the clinical samples, as per manufacturer’s instructions. 140 μL of CSF, saliva or tissue homogenates (brain, skin) were used, and RNA was eluted in 60 μL of elution buffer. All antemortem human samples, and postmortem dog saliva samples were tested with a real-time TaqMan RT-PCR targeting the RABV nucleoprotein gene, as described previously [11], 13]. PCR amplification and data analysis was performed using a BIO-RAD C1000 Thermocycler instrument and accompanying software. All samples were tested in duplicate, and samples with a mean threshold cycle (Ct) value of ≤35 were considered positive, while those with Ct between 35 and 40 were considered inconclusive, and those with no amplification were considered negative.

### LN34 pan-lyssavirus assay

For all samples, the same RNA elute was used for both the RT-PCR and the LN34 assay. Mastermix preparation, and amplification was performed using the primer and probe sequences recommended by WHO [14] and cycling conditions as per the protocol reported previously [9], with a minor modification, i.e., omission of LN34lago probe because of its limited utility in our setting. Another modification was the use of known RABV positive RNA as positive control instead of the synthetic RNA control in the original protocol. The LN34 assay was performed simultaneously with the routine RT-PCR assay. A BIO-RAD C1000 Thermocycler instrument and software was used to perform PCR amplification and data analysis. All specimens were tested in triplicate and specimens with a mean threshold cycle (Ct) value of ≤35 were considered positive, while mean Ct value of >45 was considered negative. Specimens with Ct values between 35 and 45 were considered inconclusive and the test was repeated.

### Controls for PCR

Every PCR run (for both routine RT-PCR and LN34 assays) included a positive control (RNA extracted from a known positive specimen); a non-template control (nuclease free water in place of template RNA); and an extraction control (RNA extraction performed on nuclease free water). In order to verify sample adequacy and satisfactory extraction procedure, a PCR reaction targeting beta-actin was set up for all human and animal specimens.

### Next generation sequencing (NGS)

For all samples where discordant results were obtained between two diagnostic tests, or “inconclusive” results were obtained in any PCR, next generation sequencing using the Illumina platform was performed, provided that a mean Ct value of ≤30 was obtained in at least one of the PCR assays.

### Statistical analysis

For animal and human brain samples, the results of LN34 PCR assay were compared to the reference FAT. Estimates of diagnostic accuracy and their precision were calculated using standard formulas. The performance of LN34 assay was compared with routine RT-PCR for ante-mortem human samples and dog saliva samples. The degree of agreement between the two tests was determined using Kohen’s Kappa measurement. Finally, the diagnostic performance of both the molecular tests from dog saliva samples was compared to the reference test (FAT) performed on brain samples of the same dogs, and estimates of diagnostic accuracy were calculated.

**Figure 1:**
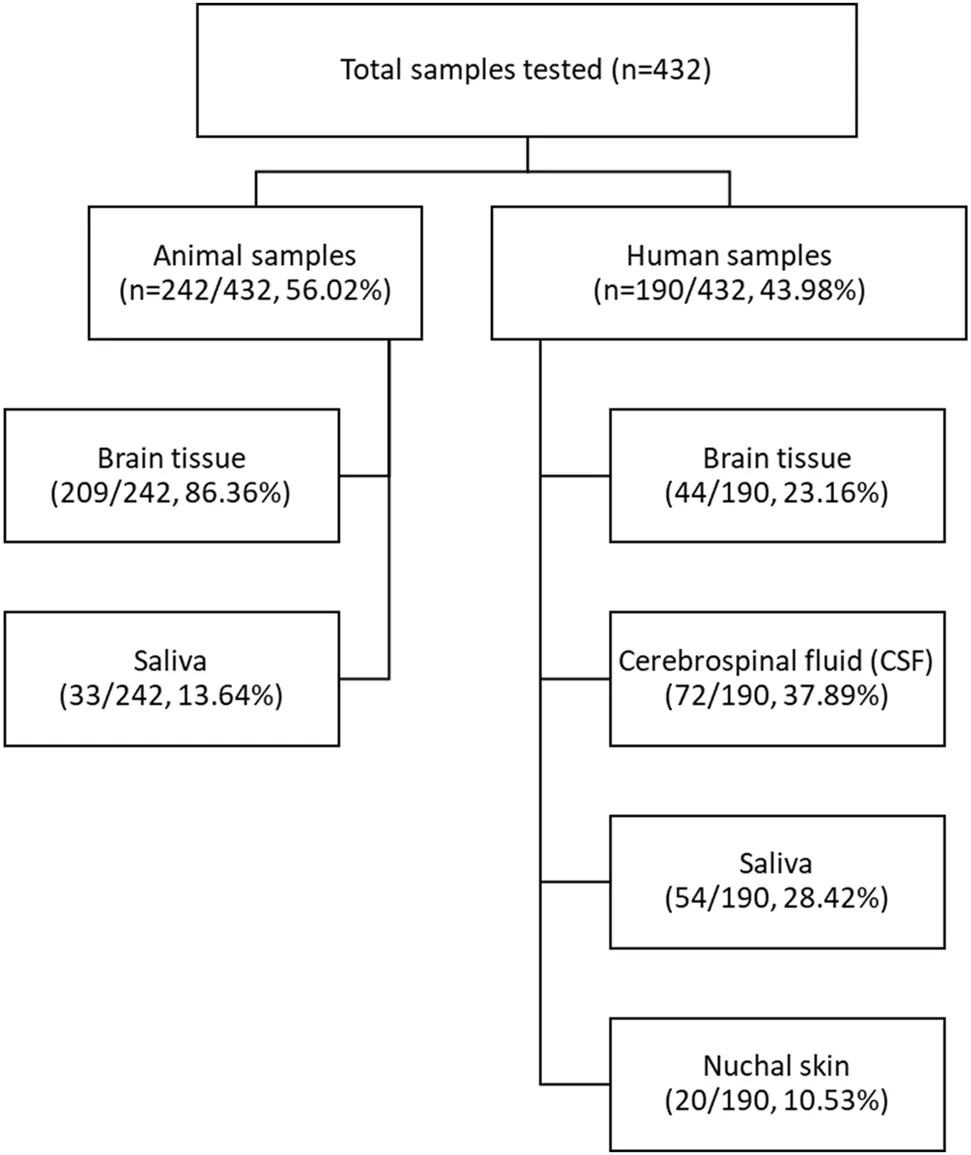
Details of human and animal samples tested in the study.

The numbers and types of human and animal samples tested in the study are summarised in Figure 1.

## Results

### Diagnostic performance of LN34 assay with animal and human brain specimens

**Figure 2:**
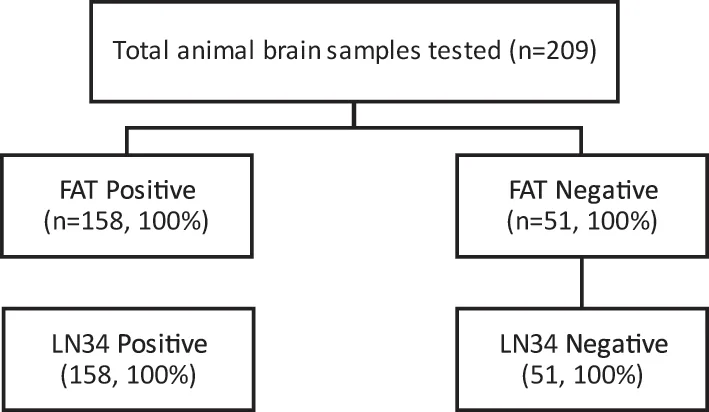
Results of animal brain samples tested with FAT and LN34 assay.

A total of 209 animal brain samples were tested with FAT and LN34 assay (Figure 2). These included 196 samples (93.78 %) from dogs, 8 (3.82 %) samples from cattle (cows and buffaloes), 2 (0.96 %) samples from jackals, and 1 (0.48 %) sample each from goat, horse, and monkey.

The results of all the samples tested are summarized in Figure 2. The beta actin control was positive (Ct <35) for all samples tested. The average Ct value of positive animal brain samples in the LN34 assay was 24.68 (range 15.7–35).

The sensitivity and specificity of the LN34 assay when compared to the gold standard FAT was 100 % (95 % CI 97.66 %–100.00 %) and 100 % (95 % CI 93.02 %–100 %) respectively. The positive predictive value was 100.00 % (95 % CI 97.66 %–100.00 %) and the negative predictive value was 100.00 % (95 % CI 93.02 %–100.00 %). Overall, the detection rate of LN34 assay and FAT for animal brain samples was 75.60 %.

**Figure 3:**
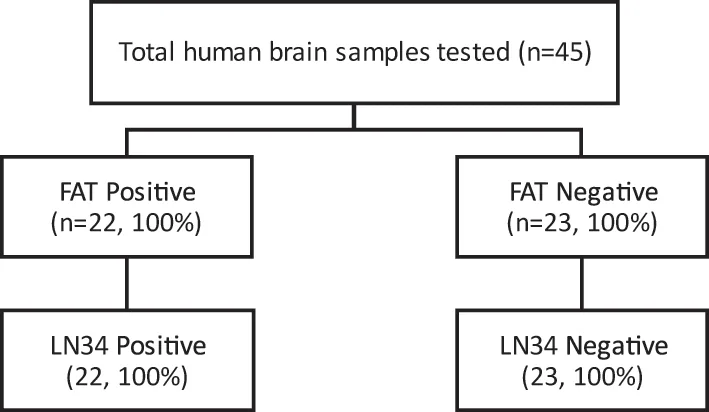
Results of human brain samples tested with FAT and LN34 assay.

A total of 45 human brain samples were tested with FAT and LN34 assay (Figure 3). The results of all the samples tested are summarized in Figure 3. The beta actin control was positive (Ct ≤35) for all samples tested. The average Ct value of positive human brain samples in the LN34 assay was 23.22 (range 14.6–34).

The sensitivity and specificity of the LN34 assay when compared to the gold standard FAT was 100 % (95 % CI 83.16 %–100 %) and 100 % (95 % CI 85.18 %–100.00 %) respectively. The positive predictive value was 100.00 % (95 % CI 83.16 %–100.00 %) and the negative predictive Value was 100.00 % (95 % CI 85.18 %–100.00 %). Overall, the detection rate of LN34 assay and FAT for human brain samples was 48.89 %.

### Diagnostic performance of LN34 assay with human antemortem specimens

**Figure 4:**
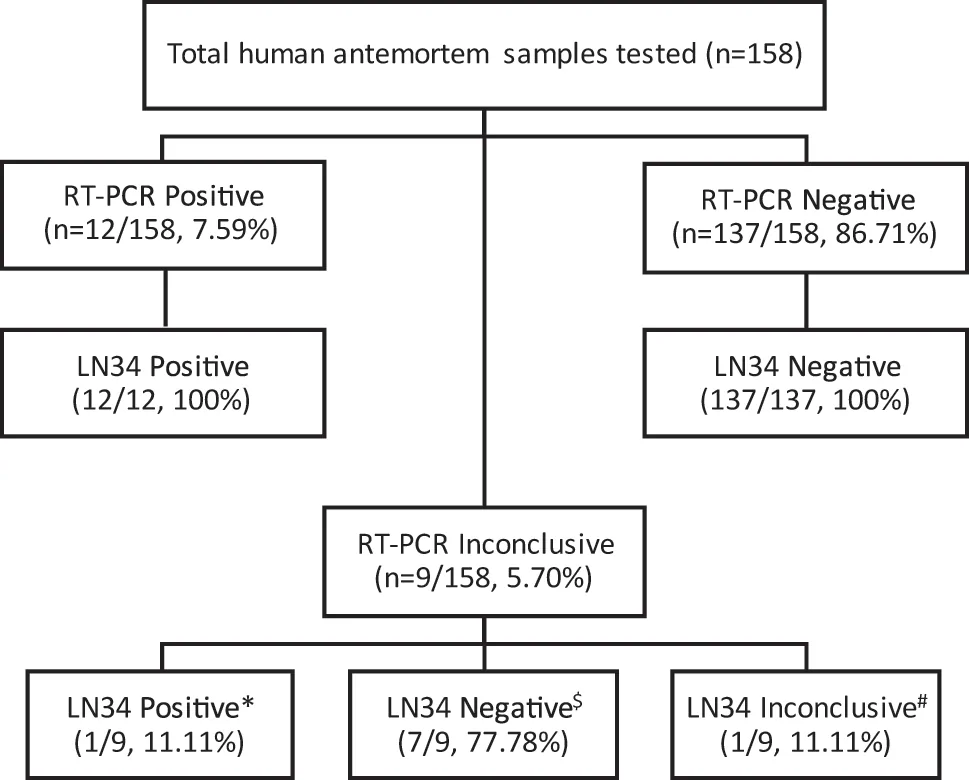
Results of human antemortem samples tested with RT-PCR and LN34 assay. *Nuchal skin sample unsuitable for NGS; ^$^3 saliva, 2 nuchal skin and 2 CSF samples, unsuitable for NGS; ^#^CSF – rabies lyssavirus detected by NGS.

A total of 158 human antemortem samples, including CSF (79, 50.00 %), saliva (59, 37.34 %), and nuchal skin (20, 12.66 %) were tested with the two molecular assays. The results are summarised in Figure 4. The beta actin control was positive (Ct ≤35) for all samples tested. All the RT-PCR positive and negative samples also tested positive and negative, respectively, by LN34 assay. Table 1 shows the average Ct value of positive human specimens in both the assays.

**Table 1:** Average and range of Ct values obtained for human antemortem samples and dog saliva samples in two molecular assays.

Sample	Average of RT-PCR Ct (range)	Average of LN34 assay Ct (range)
Dog saliva	31.08 (23.96–34.20)	32.32 (30.00–34.40)
Human CSF	30.66 (26.30–34.40)	30.40 (24.00–33.00)
Human saliva	32.32 (27.50–34.10)	32.55 (31.10–34.40)
Human nuchal skin biopsy	34.90 (single sample)	33.45 (33.00–33.90)

Out of 9 RT-PCR inconclusive samples, 1 CSF sample was also inconclusive by LN34 assay. Rabies lyssavirus was detected in this sample by NGS. Seven RT-PCR inconclusive samples (3 saliva, 2 nuchal skin and 2 CSF samples) were negative by LN34 assay and 1 sample (nuchal skin) was positive by LN34 assay. However these discordant samples were not suitable for NGS.

Out of 158 samples, 149 were included for statistical analysis, after excluding nine samples that were inconclusive with the routine RT-PCR assay. For these 149 samples, there was perfect agreement (Cohen’s Kappa equal to 1) between the routine RT-PCR and LN34 assay.

For CSF, the LN34 and RT-PCR positivity rates were 6.33 % each; for saliva, the LN34 and RT-PCR positivity rates were 10.17 % each; and for nuchal skin, LN34 positivity rate was 10 % while RT-PCR positivity rate was 5 %. Overall, the positivity rate for all antemortem specimens by LN34 assay was 8.23 % while RT-PCR positivity rate was 7.59 %.

### Diagnostic performance of LN34 assay with dog saliva specimens

**Figure 5:**
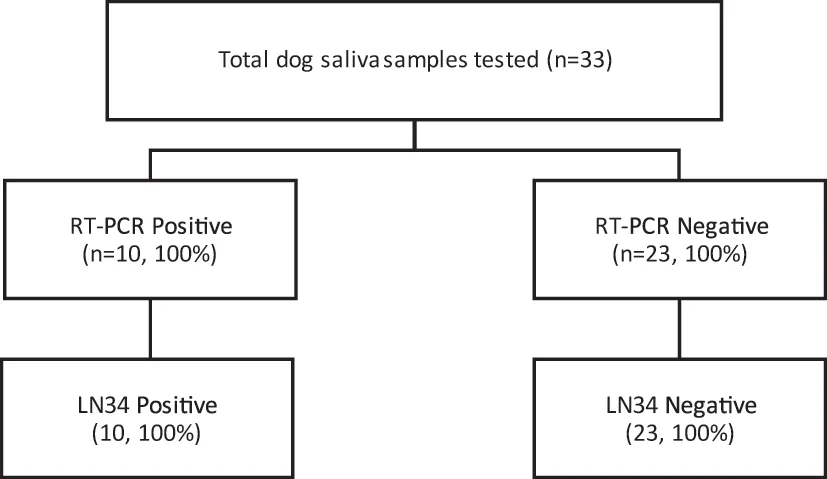
Results of dog saliva samples tested with RT-PCR and LN34 assay.

A total of 33 dog saliva samples were tested with the routine RT-PCR and LN34 assay. The results are summarised in Figure 5. The beta actin control was positive (Ct ≤35) for all samples tested. The Cohen’s Kappa for the two tests was 1.000 (95 % CI 1.0 to 1.0, SE 0.00), signifying almost perfect agreement between the two tests. The positivity rate for dog saliva with LN34 assay and routine RT-PCR was 30.30 %. Table 1 shows the average Ct value of positive dog saliva specimens in both the assays.

Furthermore, for 32 dogs whose saliva was tested, brain samples were obtained from the same dogs and FAT was performed. Out of 18 dogs with laboratory confirmed rabies, confirmation was obtained in 9 (50 %) cases with saliva RT-PCR and LN34 assays. Meanwhile, in the remaining 9 (50 %) cases, both the salivary molecular assays were negative. None of the saliva samples from FAT-confirmed negative cases additionally tested positive by either of the molecular assays.

The diagnostic performance of both RT-PCR and LN34 assay on saliva samples was calculated separately, taking the result of brain FAT as the reference standard. The sensitivity and specificity of both assays, with 32 paired samples, was 50 % (95 % CI 26 %–74 %) and 100 % (95 % CI 76.8 %–100 %) respectively.

## Discussion

The LN34 PCR assay targets a region in the N gene at the beginning of the lyssavirus genome, which is conserved across many species of lyssaviruses. The assay employs degenerate primers and locked nucleic acid probes, and it has been proven to successfully detect 88 diverse lyssavirus isolates [9]. There is substantial evidence for the robustness and reliability of this assay for detection of lyssaviruses from animal brain specimens because of the novel probe design.

During the development phase, the assay was validated with a panel of 169 FAT-confirmed animal brains and 58 additional FAT-unfit specimens, with excellent results [10]. The assay showed high diagnostic sensitivity (99.9 %) and specificity (99.68 %) in a worldwide evaluation with approximately 3,000 animal specimens [9], and near-perfect agreement with FAT for 6,855 animal specimens tested in USA [15]. The assay has also been successfully evaluated for detection of nine different RABV variants from 44 animal specimens in Argentina, and from 88 animal samples that were confirmed by direct immunofluorescence and mouse inoculation test in Brazil with 100 % detection rate reported in both studies [16], 17].

Additionally, the LN34 assay has been employed for varied and innovative applications in rabies research. For example, it was demonstrated to have high sensitivity for detection of rabies viral RNA from lateral flow assay strips stored in a refrigerator or at room temperature (96.2 % and 100 %, respectively) [18]. A mobile PCR device, i.e., the PicoGene PCR1100 instrument, which utilizes the pan-lyssavirus LN34 primer system has also been evaluated for diagnosis of rabies in the Philippines. The sensitivity and specificity of this assay was reported to be 100 % when tested with canine and feline brain specimens obtained from the field [19]. A modified protocol, named “(n)LN34 assay”, has also been developed, and showed higher sensitivity for the rabies lyssavirus and other lyssaviruses [20]. The LN34 assay, followed by genomic sequencing, was instrumental in the discovery of a novel bat-associated lyssavirus, i.e., the Divača bat lyssavirus, in Slovenia as a part of a national retrospective surveillance programme [21].

Hence it is evident that the LN34 assay is a robust and well-validated diagnostic modality for laboratory confirmation of rabies. The original validation panel included 37 human brain specimens [10], and another study from the Dominical Republic describes the use of LN34 assay for detection of RABV from a single formalin-fixed human brain specimen [22].

Antemortem rabies diagnosis is vital for clinical trials of new treatments, confirming human cases where postmortem brain samples are hard to obtain, and distinguishing atypical rabies from similar conditions. Rapid lab results also enable prompt post-exposure care and infection control.

Evidence describing the use of LN34 assay in antemortem specimens is exceedingly limited. Case reports from Texas, USA and Qatar have described the detection of rabies viral RNA from antemortem specimens such as nuchal skin biopsy [23] and saliva [23], 24]. But other reports have described cases where although history was suggestive of rabies, antemortem samples tested negative by LN34 assay, and diagnosis was ultimately confirmed by other techniques, i.e., antemortem detection of neutralizing antibodies in serum and CSF [25], and postmortem detection of viral antigens and RNA in brain tissue [25], 26].

For human antemortem samples, there was complete concordance between the RT-PCR and LN34 assay for the positive and negative samples, however there were nine inconclusive samples out of which only one could be resolved by NGS. These samples represent the limitations of antemortem sampling for testing of rabies and underscore the necessity of serial or multiple sampling for antemortem confirmation of rabies. Since these specimens were unsuitable for NGS, further evaluation with metagenomic sequencing is required for the samples that tested positive with LN34 assay but inconclusive with the routine RT-PCR. Diagnostic confirmation may be obtained in these patients by testing for neutralizing antibodies in serum and/or CSF, although these tests carry their own caveats.

Overall, LN34 assay can complement the tests currently being used in our setting for antemortem confirmation of rabies. Additionally, LN34 assay is valuable for a reference laboratory such as ours for continuous surveillance to detect the possible emergence of non-rabies lyssaviruses in the future, since these have so far not been reported from India. The testing of wildlife samples is especially important for this purpose.

An important aspect of this study was the evaluation of postmortem dog saliva as a sample for routine rabies testing and surveillance by the LN34 assay. This is significant due to the ease of obtaining this sample, as compared to the currently prevailing standard sample, i.e., brain tissue. Canine saliva has been previously studied as a sample for detection of rabies viral RNA by a PCR targeting the N gene, and a sensitivity of 84.6 % was reported from confirmed dog rabies cases [27].

While our study found that postmortem dog saliva has low sensitivity for rabies detection, its perfect specificity is valuable for veterinary surveillance, especially where collecting and transporting brain samples or performing FAT is challenging. A positive oral swab means necropsy or brain tissue collection is unnecessary. Saliva sampling is simple, and with increasing access to molecular diagnostics, this method warrants further large-scale evaluation and potential field use. However, decisions related to post-exposure prophylaxis in humans must never rely on, or be delayed due to canine saliva testing results.

The LN34 assay has consistently shown strong diagnostic performance for rabies in both previous and current studies. Its main strength is its ability to detect both rabies and non-rabies lyssaviruses at the same time. However, this feature has limited relevance in India, as there is currently no evidence of non-rabies lyssaviruses or bat-mediated rabies [3]. Additionally, the high cost of the LN34 assay compared to other real-time RT-PCR assays restricts its use as a routine diagnostic tool. Nonetheless, it remains suitable for reference laboratories or advanced centers that monitor non-rabies lyssaviruses.
